# Minimally Invasive Successful Reconstruction of a Severely Traumatized Upper Extremity Using Platelet-Rich Plasma and Tissue Scaffold: A Case Report

**DOI:** 10.1055/s-0041-1742176

**Published:** 2022-01-17

**Authors:** Srinjoy Saha

**Affiliations:** 1 Department of Plastic Surgery, Apollo Multispecialty Hospital, Kolkata, West Bengal, India

**Keywords:** debridement/methods, regenerative medicine, reconstructive surgical procedures/methods, soft tissue injuries/surgery, upper extremity/injuries

## Abstract

Minimally invasive reconstruction combines principles of tissue engineering and regenerative medicine for healing complex wounds. This approach was successfully demonstrated on a 64-year-old diabetic and hypertensive male patient, who was brought unconscious to our emergency after surviving an automobile collision with severe brain and right-dominant upper extremity injuries. Uncontrolled hyperglycemia, severe anemia, diffuse axonal brain injury, wrist drop, and loss of thumb extension and abduction were noted. Extensive degloving, skin necrosis, extensor and flexor forearm muscle crush injuries, and ruptured extensor tendons were observed. Serial wound debridement combined with platelet-poor plasma injection into the muscles, platelet-rich plasma injections into the tendons and subcutis, and low-negative pressure wound therapy were performed sequentially to salvage the injured soft-tissues. Improvements were noticed during the second exploration after 5 days. Surviving muscles showed adequate vascularization and revival of innervation during the third exploration after another 5 days. Thereafter, absorbable synthetic tissue scaffold was applied over a sizeable 270 cm ^2^ wound as a flap-alternative. Tissues regenerated well within the scaffold during the next 2 months, halving the wound area to 132 cm ^2^ . A thick split-skin graft was applied over the remaining granulating neodermis, which “took” completely. Six months postoperatively, the patient regained most hand functions and performed all activities satisfactorily, while the grafted area appeared almost identical to surroundings. Minimally invasive reconstruction thus produced satisfying results with fewer shorter simpler surgeries, minimal anesthesia, short-duration hospitalization, lower health care costs, lesser risks, and excellent patient-reported outcomes.

High-velocity automobile accidents cause massive skin, muscle, and tendon injuries in the traumatized extremities, often requiring amputation or several extensive surgeries, with disability persisting in some patients. In high-velocity collision, greater tissue damage occurs than it is initially apparent, while progressing “zone of injury” produces progressive necrosis. Earlier, for managing such injuries, serial debridement used to be performed every 48 hours, debriding definitely necrosed and preserving questionably viable tissues. However, this approach was prone to infections, required multiple surgeries, and lengthy hospitalization. Currently, wound excision or immediate radical debridement is recommended. All nonviable and suspicious tissues are debrided, leaving only those tissues which are clearly alive. 1 It results in substantial tissue loss, thus necessitating reconstruction of tendons, arteries, and nerves later for restoring functionality. 2 Finally, the resulting extensive wound requires substantial reconstruction with well-vascularized flaps. 1 2 Choices for wound reconstruction includes fasciocutaneous flaps, pedicled abdominal flaps, and microvascular free tissue transfers. 2 3 4 Negative-pressure wound therapy (NPWT) and biomaterial application followed by skin grafting are recent additions. 2 5

Striking a middle ground between these two approaches, minimally invasive reconstruction focuses on accurate conservative debridement performed every 5 days. Regenerative therapy is combined to prevent necrosis of soft tissues with suspicious viability while simultaneously guarding against infections. Strengthening the surviving muscles and tendons with early physiotherapy maximizes their function, thereby obviating the need for significant reconstructions later. After achieving a healthy wound, tissue scaffolds are applied and covered with skin graft afterward as an alternative to more extensive flap surgeries. This method was successfully demonstrated on a severely traumatized upper extremity of an elderly diabetic patient presenting with hemorrhagic and neurogenic shock, and their outcomes documented 9 months postoperatively.

## Case Presentation

### History

**Fig. 1:**
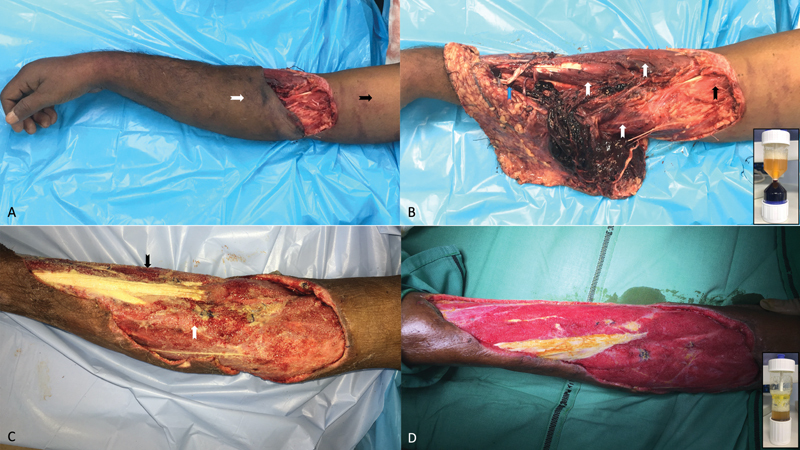
the injured upper limb. ( **A** ) An elderly diabetic man presented with severely traumatized upper extremity. Degloved skin was discolored at presentation (white arrow) compared with healthy skin (black arrow). Wrist drop was evident. ( **B** ) Massively injured extensor and flexor forearm muscles were grossly discolored (white arrows). Proximal flexor muscles appeared healthy in comparison (black arrow). Some extensor tendons were ruptured (blue arrow). (Inset, bottom right) Platelet-rich plasma (yellowish fraction) was obtained by centrifuging venous blood. ( **C** ) Reexploration 5 days after regenerative bioengineered approach showed visible improvements in surviving muscles, which now appeared pale to pinkish. Muscles injured more were darker (black arrow) compared with less-injured muscles, which appeared pinkish (white arrow). ( **D** ) Third exploration after another 5 days showed a well-vascularized mass of surviving muscles, which bled on cuts and responded to electrocautery. Some tendons appeared healthier after plasma injections. (Inset, bottom right) Platelet-poor (light yellow, upper) and platelet-rich (amber-colored, lower) plasma fractions were obtained after recentrifugation of the first platelet-rich plasma sample.

A 64-year-old diabetic and hypertensive man was brought unconscious to our emergency after a high-velocity automobile accident. He suffered from massive bleeding, extensive brain injuries, and expansive soft tissue injuries over his right-dominant upper extremity. At presentation, we noted severe anemia with hemoglobin at 5.6 g/dL, uncontrolled blood glucose at 535 g/dL, substantial forearm degloving with skin necrosis, extensive muscle injuries, and multiple tendon disruptions ( Fig. 1A ). Computed tomogram of the brain diagnosed diffuse axonal injury.

### Examination

After the patient was stabilized in our neurosurgical intensive care, wrist drop along with loss of thumb extension and abduction were observed, and the viability of discolored degloved skin appeared doubtful. During the first surgical exploration, the degloved skin paddle had a narrow distal pedicle, appeared dark and contused, with traumatized subcutaneous fat and scanty dark venous oozing on dermal incisions ( Fig. 1A, B ). Muscle bulk of all the forearm extensors and some of the flexors appeared discolored, neither bleeding on cuts, nor responding to electrocautery ( Fig. 1B , white arrows). Comparatively, other noninjured flexor muscles appeared healthy ( Fig. 1B , black arrow). While the finger extensors were intact, both the wrist extensors, thumb extensor, and thumb abductor had avulsed off ( Fig. 1B , blue arrow).

### Tissue Regeneration

Attempting maximal salvage of the crushed deep tissues, accurate conservative debridement preserving doubtful muscles and tendons was performed, thereby contradicting current recommendations of radical wound excision. Platelet-concentrate injections and NPWT followed thereafter. Briefly, 27 mL of venous blood was mixed with 3-mL sodium citrate and centrifuged twice ( Fig. 1B , inset). Two cycles of centrifugation produced 6-mL light-yellow platelet-poor plasma (PPP) and 6-ml amber-colored platelet-rich plasma (PRP; Fig. 1D , inset). PPP was injected into the injured skeletal muscles and PRP into the injured tendons and subcutis. Finally, NPWT was applied at 75 mm Hg, and the process repeated during each surgical exploration.

### Soft Tissue Salvage

Second surgical exploration after 5 days revealed pale/pinkish muscles and yellowish tendons ( Fig. 1C ). Absence of progressive necrosis and infection within the doubtful muscles was observed. Less-injured flexor muscle bulk appeared pinkish ( Fig. 1C , white arrow), while the more-injured extensor muscle bulk appeared darker ( Fig. 1C , black arrow). Significant areas of the degloved skin were found to be devascularized, with no bleeding on dermal incisions and were excised completely. Third exploration after another 5 days revealed survival of all the remaining muscle mass which appeared reddish and viable ( Fig. 1D ). Now, they bled on cuts and responded weakly to electrocautery, and muscle biopsies confirmed presence of healthy muscle tissues.

### Tissue Scaffold Application

**Fig. 2:**
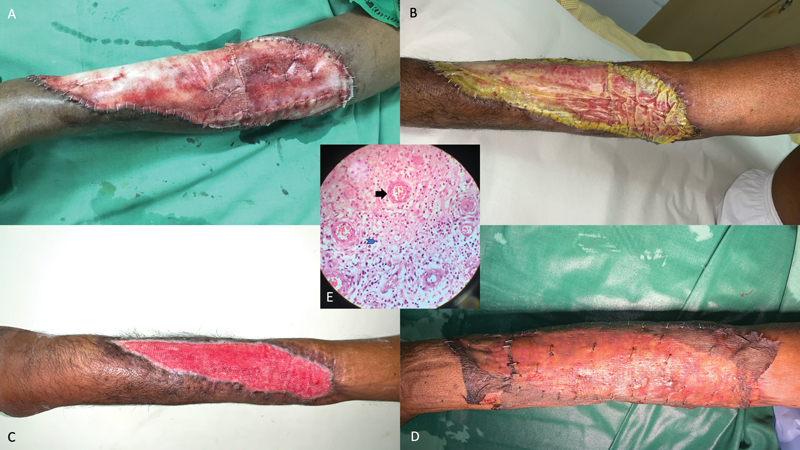
Biomaterial application over the sizeable wound. ( **A** ) Significant wound sized approximately 27 cm × 10 cm was covered with a synthetic biomaterial. ( **B** ) One month later, shortening of the wound size was observed through the translucent outer film with neodermis forming within the inner foam matrix. ( **C** ) Two months later, significant tissue regeneration was evident within the biomaterial. Wound dimensions now measured 22 cm × 6 cm, halving the total area from 270 to 132 cm ^2^ . ( **D** ) A thick split-skin graft applied over the lush granulating neodermis “took” completely. ( **E** ) Histopathological examination of granulating neodermis revealed budding new blood vessels (black arrow) and neutrophil infiltration (blue arrow) within the biomaterial.

During preoperative counseling, the patient insisted on avoiding both long-duration reconstructive surgeries and general anesthesia, fearing his existing brain injury. Accordingly, after injecting platelet concentrates, the sizeable 27 cm × 10 cm wound was covered with a bilayered polyurethane tissue scaffold (Biodegradable temporizing matrix, Polynovo Ltd, Melbourne, Australia) under sedation and local anesthesia, and the patient discharged the next morning ( Fig. 2A ). Regular physiotherapy was performed at home to strengthen the surviving muscles and tendons, even as neodermis regenerated within the inner foam layer of the applied scaffold. Dressings were changed weekly.

### Initial Results

After 1 month, progressive tissue regeneration within the biomaterial was evident through the translucent outer film on top ( Fig. 2B ). After 2 months, delamination of the outer film revealed exuberantly granulating neodermis over the exposed wound. Wound dimensions had shortened significantly, from 27 cm × 10 cm initially to 22 cm × 6 cm eventually, halving the total wound area from 270 to 132 cm ^2^ ( Fig. 2C ). Biopsy from the granulations within the neodermis showed neutrophil infiltration ( Fig. 2E , blue arrow) and budding new blood vessels ( Fig. 2E , black arrow). The remaining granulating neodermis was covered with a thick split-skin graft which “took” completely within a week ( Fig. 2D ).

### Final Results

**Fig. 3:**
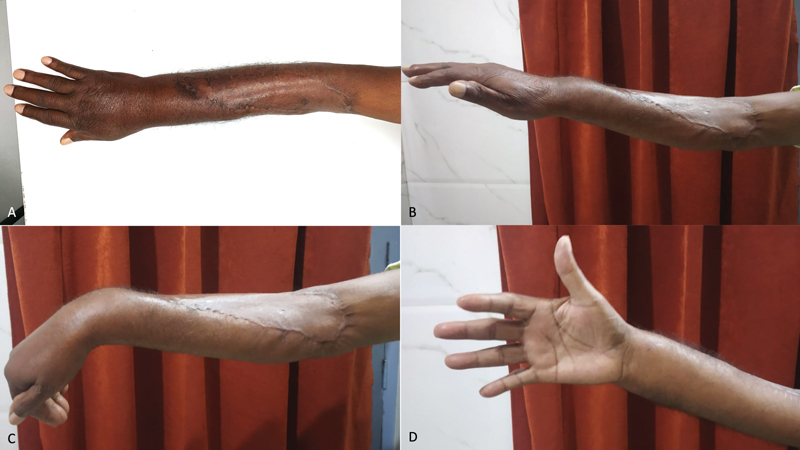
Postoperative results after 9 months. ( **A** ) The skin-grafted wound appeared nearly identical to the surrounding healthy areas. ( **B** ) Wrist drop and finger drop were absent, with an acceptable wrist extension of 15 degrees. ( **C** ) Wrist and finger flexion movements were satisfactory, with an acceptable wrist flexion of 50 degrees. ( **D** ) Thumb abduction and extension were satisfactory, allowing all functions.

Postoperative recovery was uneventful. The patient needed support for posttraumatic stress disorder, and reluctantly performed physiotherapy. Six months postoperatively, excellent pliability and appearance of the grafted wound were apparent ( Fig. 3A ). The patient had good sensation, adequate range of finger movements, no wrist drop, acceptable active wrist extension of 15 degrees, adequate active wrist flexion of 50 degrees, full thumb abduction, and adequate thumb extension ( Fig. 3B–D ). Muscle power was four-fifths for finger extension, thumb abduction, and extension, and three-fifths for wrist extension.

### Outcomes Assessment

Motivating this patient for physiotherapy became difficult as he was suffering from posttraumatic stress disorder. Ideally, better physiotherapy would have achieved better power of the surviving muscles and tendons, and better motion over his hand joints. Practically, however, he would only perform the bare minimum exercises necessary to enable an active independent lifestyle and was reluctant to exercise otherwise. During postoperative outcomes, assessment after 9 months, he considered the results to be excellent ( Table 1 ). He was immensely satisfied after regaining the necessary hand functions and adequately performing all routine work and the activities he was passionate about, including writing, gardening, and carpentry.

**Table 1:** Patient-reported outcomes 9 months after surgery

Patient's responses to questions on a	Visual analogue scale
How well did this treatment solve your problem?	10/10
How pain free was your entire surgical experience?	9/10
How well do you perform your regular daily work now?	10/10
How well do you perform strenuous activities now?	9/10
How satisfied were you with the treatment approach?	10/10
How good and skillful do you think your surgeon was?	10/10
If necessary, would you repeat this approach in future?	10/10
Would you recommend this approach to someone close?	10/10

## Discussion

Radical wound excision of all necrosing, nonbleeding, and unresponsive muscles, followed by NPWT at 125 mm Hg, is the current mainstay for treating massive soft tissue injuries, thereby resulting in significant loss of muscles and tendons. 1 2 After the initial wound stabilization, fasciocutaneous or free-flap reconstruction, tendon reconstructions, tendon transfers, and other flap modifications are necessary to restore form and function of the injured extremity. 2 4 Existing reconstructive approach thus requires multiple significant surgeries and an expensive setup, thereby increasing time, costs, and morbidity.

Contrastingly, accurate serial debridement provides injured muscles and tendons a chance to vascularize and function again. Only the definitely necrotic muscle tissues are debrided, as apparent from their completely black color and shriveled appearance. All other doubtful tendon and muscles, including those not bleeding to cuts nor responding to electrocautery, are preserved and serially treated with different platelet concentrates and NPWT until they become definitely healthy, or else, fully necrosed.

Reviving massively injured muscles (appearing dark, nonbleeding, and noninnervated) with regenerative therapies as seen here, can be a game changer ( Fig. 1B, D ). Halting progressive tissue necrosis while avoiding infections during the second exploration was an early indicator of treatment efficacy ( Fig. 1C ). Along with platelet-concentrate injections, low NPWT at 75 mm Hg played a significant role in tissue salvage. Earlier studies have shown that low NPWT sufficiently increased the blood flow within ischemic muscles while protecting the poorly perfused tissues. It was reported that NPWT at 125 mm Hg does not adequately protect these injured ischemic muscles. 6 7 Sealed dressings of NPWT maintains sterility of the wound site and minimizes the chances of acquiring nosocomial infections in the wards.

Growth factors play an important role in muscle regeneration after injuries. Platelet-derived growth factor, basic fibroblast growth factor, and hepatocyte growth factors stimulate satellite cells in the muscles, resulting in myoblast proliferation. Insulin-like growth factor-1 stimulates both differentiation and proliferation of myoblasts. Vascular endothelial growth factor enhances repair by increasing angiogenesis. PRP contains several of these growth factors in the α granules of platelets which slowly released over a week. 8 Platelet-rich fibrin is a modification that does not require the addition of any anticoagulants in the blood. It contains platelets, leucocytes, cytokines, and many adhesive proteins, forming a cross-linked matrix that slowly releases growth factors from platelets and leucocytes, thereby promoting tissue regeneration. 9

In previous preclinical studies, PPP greatly benefitted injured muscles by inducing myoblasts into muscle differentiation, becoming crucial for new muscle production. 10 PRP induced myoblast proliferation, but not differentiation, and treated tendinopathies and tendon injuries more efficiently. 10 11 Growth factors and cytokines released by a concentrated pool of activated platelets contributed to the modulation, angiogenesis, and immune response of inflammation, thereby promoting healing and repair. 11 A three- to five-fold increase in growth factors were observed in PRP compared with nonconcentrated whole blood. 12

Tissue scaffolds offered an alternative, simpler, and less-invasive way of reconstructing severe wounds with minimum morbidity than reconstructive flaps. 5 13 Commercially available tissue scaffolds include biological and synthetic varieties, and all have been reported to be efficacious. 13 The bilayered synthetic tissue scaffold used here comprises of an inner 2-mm-thick absorbable polyurethane foam, covered on the outside with a semipermeable polyurethane film. The inner foam layer degrades over time by hydrolysis, even as fibroblasts and new blood vessels infiltrate and proliferate within it, forming a neodermis. Earlier, when PRP was combined with a tissue scaffold, it reliably covered sizeable areas of exposed avascular skull bones with robust granulation cover, without requiring immobilization or NPWT. 14

Preventing a disability is a far more efficient form of treatment than correcting one. Being more patient friendly, minimally invasive reconstruction can immensely benefit our society by increasing the effectiveness and quality of limb salvage reconstructions while lowering costs and reducing morbidity. Since simpler instruments in smaller setups are sufficient for this approach, it would decrease health care investment costs substantially. In spite of the many advantages observed with this approach in this case report, to rule out chance and confounding factors, a well-designed large study would be necessary to validate its effectiveness in future.

## Conclusion

Minimally invasive reconstruction comprising of spaced-out serial debridement, platelet-concentrate injections, low-negative suction, and biomaterial application (with skin-grafting later) possesses several advantages. It simplifies surgeries, reduces tissue loss, minimizes anesthesia, shortens hospitalization, rehabilitates early, and lowers overall health care costs. In this case report, describing a severely traumatized upper extremity in an elderly diabetic patient who was notoriously difficult to heal, this approach enabled successful reconstruction and documented excellent patient-reported outcomes.
